# Measuring What Matters Across International Long-Term Care Settings

**DOI:** 10.1093/geroni/igab046.609

**Published:** 2021-12-17

**Authors:** Michael Lepore, Kirsten Corazzini

**Affiliations:** 1 LiveWell Alliance, Southington, Connecticut, United States; 2 University of Maryland School of Nursing, Baltimore, Maryland, United States

## Abstract

Measuring what matters most to residents, relatives and staff in residential long-term care settings is critical, yet underdeveloped in our predominantly frailty and deficits-focused measurement frameworks. The Worldwide Elements to Harmonize Research in Long-Term Care Living Environments (WE-THRIVE) consortium has previously prioritized measurement concepts in the areas of care outcomes, workforce and staffing, person-centered care, and care context. These concepts include knowing the resident and what matters most to the resident, and outcomes such as quality of life, and personhood. We present findings of our currently recommended measures, including both general population and dementia-specific measures, such as the Person-Centered Care Assessment Tool (PCAT), the Personhood in Dementia Questionnaire (PDQ), and the ICEpop CAPability Measure for Older People (ICECAP-O). We also describe remaining gaps in existing measures that will need to be addressed to fully specify common data elements focused on measuring what matters most to residents, relatives and staff.

